# Influence of flowering on the anatomical structure, chemical components and carbohydrate metabolism of *Bambusa tuldoides* culms at different ages

**DOI:** 10.3389/fpls.2023.1260302

**Published:** 2023-11-03

**Authors:** Jiaxin Liu, Yufang Wu, Li Zhou, Anmian Zhang, Sushuang Wang, Yi Liu, Dejia Yang, Shuguang Wang

**Affiliations:** ^1^ Faculty of Life Sciences, Southwest Forestry University, Kunming, China; ^2^ Faculty of Bamboo and Rattan, Southwest Forestry University, Kunming, China; ^3^ Key Laboratory for Forest Resources Conservation and Use in the Southwest Mountains of China, Ministry of Education, Southwest Forestry University, Kunming, China

**Keywords:** *Bambusa tuldoides*, flowering culms, anatomical structure, chemical properties, sugar metabolism, utilization

## Abstract

Bamboo forests, which have come to occupy large areas in recent years, naturally undergo the process of blooming. However, bamboo culms and rhizomes degenerate after the plants bloom, resulting in widespread loss of raw materials. Systematic research on the properties and physiology of bamboo culms after flowering is lacking, and whether flowering bamboo culms could be used as raw materials in industry is unclear. In this paper, we compared and measured the fiber morphology, chemical components, and sugar metabolism indexes of non-flowering and flowering *Bambusa tuldoides* culms at different ages. The results showed that the fibers in the middle internodes of both non-flowering and flowering *B. tuldoides* culms had the longest length. The fibers completed their elongation within 1 year, but the fiber walls were continually deposited with age. The levels of the chemical components in the nonflowering culms also continually increased with age. The nonstructural carbohydrate (NSC) content and sugar metabolism indexes showed the highest levels in the 2-year culms and then declined in the 3-year culms. Compared to young culms that had not yet flowered, the 3-month-old and 1-year-old flowering culms had a significant decrease in the fiber length and tangential diameter, and their holocellulose and lignin levels also decreased, while the levels of ash, SiO_2_, 1% NaOH extractives, and benzene-ethanol extractives increased. A correlation analysis showed that sugar catabolism was accelerated in the flowering cluster, which could lead to “starvation death” in bamboo and which had a significant negative impact on the anatomical and chemical properties of the bamboo culms. Generally, the flowering bamboo culms had shorter fibers, higher levels of extractives and ash, and lower holocellulose content, which indicated that bamboo flowering has an adverse effect on the application of such components in the production of pulp, in papermaking, and in other processing and utilization activities. This study revealed the physiological changes in flowering *B. tuldoides* culms and provided a theoretical basis to inform the utilization of culms in this species.

## Introduction

Bamboo is widely distributed in subtropical and tropical regions of Asia, Africa, and Latin America (Zhou et al., 2011). The total area of various bamboo stands reaches up to 22.0 × 10^6^ ha, accounting for approximately 1.0% of the total area of global forests (Guo et al., 2005). China is the country with the richest bamboo resources in the world, with over 500 species of 39 genera (Zhou et al., 2005). Bamboo has the advantages of wide distribution, rapid growth, and renewability, making it an environmentally beneficial resource (Liu et al., 2018). The bamboo industry provides food and building materials for 2 billion people in the world and increases their incomes. Bamboo products such as bamboo shoots, furniture, charcoal, and cosmetics are used and traded by half the world’s population (Melese, 2020).

In the southern areas of China, bamboo is an important forest resource and an important source of income for local people. Bamboo possesses the advantages of a short vegetative cycle, a wide range of use, and excellent performance, and is an ideal replacement for wood (Yang et al., 2004). Bamboo is extensively used as a raw material for musical instruments, traditional handicrafts, and light-framed constructions (Maulana et al., 2020). Bamboo can also be used as a raw material for pulp (Fatriasari and Hermiati, 2008), nanocellulose (Jang, 2015), plywood (Jasni and Sulastiningsih, 2005; Suryana et al., 2011), bamboo-oriented strand board (Fatrawana et al., 2019; Maulana et al., 2019), bamboo zephyr boards (Gopar and Sudiyani, 2004) and other biomaterials and composite materials.

Bamboo is an excellent raw material for pulping due to the abundant fibers in culms. With the maturation of bamboo culms, the fibers will complete the growth of length and tangential diameter, but their walls are continuously thickened with age to form a polylamellate structure (Wang et al., 2011). Chemical property is one of the essential properties that affect the material properties and processing of bamboo. The chemical composition of bamboo is similar to that of wood, mainly containing cellulose, hemicellulose, and lignin, which account for more than 90% of the total mass (Li et al., 2007), subsequently followed by soluble sugar, resin, wax, ash, and so on (Maulana et al., 2020).

Non-structural carbohydrate (NSC) is mainly composed of soluble sugar and starch, and the soluble sugar is the product of photosynthesis (Dietze et al., 2014), and starch is the main form of energy storage (Hartmann and Trumbore, 2016). Therefore, the change in NSC content directly reflects the relationship between C-gain and C-loss (Richardson et al., 2015; Yang et al., 2016). NSC is also the key regulator of plants to resist external adverse environmental stresses, providing the energy and carbon sources to help plants maintain life activities (Zhang et al., 2013; O’Brien et al., 2014). However, these advantages will no longer exist after bamboo blooming, because bamboo usually dies after flowering (Zheng et al., 2017).

In the plant kingdom, flowering is one of the indispensable and most important processes for transitioning from the vegetative stage to the reproductive stage (Jiao et al., 2019). Most bamboos remained in the vegetative stage for decades, followed by a large-scale synchronous flowering period until death (Janzen, 1976). Bamboos consume a large amount of energy in the process of flowering, and meanwhile, the bamboo culms and rhizomes quickly degenerate after flowering and fruiting. This further leads to the death of the entire plant, which is a devastating blow to the utilization of bamboo resources and causes huge economic and ecological losses (Liese, 1987; Sertse et al., 2011). Therefore, understanding the anatomical structure and physiological and biochemical changes of bamboo culms after flowering is not only important for biological research, but also for the bamboo industry.

*Bambusa tuldoides* is mainly distributed in Guangdong, Guangxi, Guizhou, Fujian and Yunnan, and its culms are used for construction, furniture, agricultural tools, and other materials (Yi and Shi, 2008). As far as we know, most works are mainly focused on the molecular mechanism of bamboo flowering, but the studies on the physiological effects of flowering on the development and material properties of bamboo culms are few. NSC is an essential carbon source for bamboo flowering. Therefore, it is necessary to analyze the dynamic changes in sugar metabolism during flowering. In this paper, the morphological characteristics of fibers, chemical composition and sugar metabolism were analyzed in the *B. tuldoides* culms with age, and the physiological influences of flowering on culms of different ages were also determined, so as to reveal whether the flowering decreased the quality of bamboo culms. This was helpful for us to understand the physiological changes in bamboo culms during flowering and to confirm whether the flowering bamboo culms were still useful for the processing industry.

## Materials and methods

### Plant materials

The flowering and non-flowering *B. tuldoides* culms of different ages were obtained from the bamboo garden of Southwest Forestry University in Kunming, Yunnan Province in November 2021, and the bamboo entered into the flowering period in 2019. The culm age was determined according to the bamboo sheath and surface color of the culms. Four age classes (3 months, 1, 2, and 3 years old) of non-flowering and flowering bamboos were selected, and their diameters at breast height (DBH) and at culm bottom (DBC) were measured. The sampling culms were divided into three parts, i.e., bottom, middle and top, which were the 3rd, 8th and 13th part of the culms, respectively.

### Methods

#### Morphological observations on vascular bundle

A total of 24 culms were selected, including three non-flowering and three flowering culms from each age class with similar size. For each bamboo culm, a total of 9 samples were collected from the middle part of the 3rd, 8th, and 13th internodes (three samples per internode). The number of internodes was counted from the ground. The samples were cut into blocks (4cm × 1cm × wall-thickness), and then were fixed in FAA (45% ethanol, 0.25% glacial acetic acid, and 1.85% formaldehyde). After 24 hours of fixation, the samples were placed in a softener (50% glycerol, 50% ethanol) for softening treatment. The softened samples were soaked in polyethylene glycol 6000 at 60°C for one week. Subsequently, the samples were cut into sections with 20 μm of thickness by a rotary microtome (Leica RM 2165, Germany). A total of 15 sections of each sample were used for observation on vascular bundle, stained with safranin O and alcian blue, and were observed and photographed by two-dimensional measurement software (DS-3000, Caikang, Shanghai, China) under a light microscope (PH100-3B41L-IPL, Phenix, Jiangxi, China) according to the methods of Huang and Li (2023).

#### Starch grain localization

The localization of starch grains in bamboo culms was carried out by the Periodic Acid-Schiff Stain (PAS) reaction (Chu, 1963). A total of 15 sections of each sample were used for observation of starch grain. The slices were soaked in 0.5% KIO_4_ for 10 min, Schiff’s reagent for 30 min, and then dehydrated in graded ethanol. The distribution of starch grains was observed by two-dimensional measurement software under the microscope.

#### Determination of fiber morphology

The samples obtained from the 3rd, 8th, and 13th internodes of three non-flowering and flowering culms were cut into about 2 cm × 2 mm strips and macerated in Jeffery’s solution for 36-72 h, containing 10% chromic acid and 10% nitric acid (Jeffrey, 1917). After washed with distilled water to neutralize, the macerated fibers were placed in 70% ethanol for preservation. Each test included three replicates with 50 fibers in each replicate and a total of 150 fibers were measured for each internode. The length, wall thickness, and lumen diameter of fibers were recorded by two-dimensional measurement software. The tangential diameter (tangential diameter = 2 × wall thickness + lumen diameter), slenderness ratio (L/T = length/tangential diameter) and Runkel ratio [W/Lu = (2 × wall thickness)/lumen diameter] were calculated.

#### Determination of chemical properties content

The samples were placed at 105°C for 30 min, oven-dried to constant weight at 60°C, and the moisture content (MC) was calculated as the following formula: MC= (fresh weight - dry weight)/fresh weight × 100%.

The samples were oven-dried at 60°C for 24 h and then ground with a Wiley mill, followed by filtering with NO. 40 mesh and NO. 60 mesh sieves, and the residues on the NO. 60 mesh sieves were used for the subsequent chemical analyses. Chemical analyses were conducted based on the Chinese National Standards (**Table 1**). Each determination was repeated three times.

**Table 1:** Methods used for the analysis of major chemical components.

Chemical	Replicates	Standard
Ash	3	Chinese National Standard for Testing and Materials (CNSTM) (1993a)
Silicon dioxide (SiO_2_)	3	Chinese National Standard for Testing and Materials (CNSTM) (1987)
1% NaOH extractives	3	Chinese National Standard for Testing and Materials (CNSTM) (1993b)
Benzene-ethanol extractives	3	Chinese National Standard for Testing and Materials (CNSTM) (2009a)
Lignin	3	Chinese National Standard for Testing and Materials (CNSTM) (2009b)
		Chinese National Standard for Testing and Materials (CNSTM) (1995)
Holocellulose	3	Chinese National Standard for Testing and Materials (CNSTM) (1996)

#### Determination of soluble sugar, starch and NSC content

The contents of soluble sugar and starch were determined by the phenol-sulfuric acid method (Okahisa et al., 2006). The specimens (0.5 g) were ground in a mortar with liquid nitrogen for full grinding, and then were transferred into 15 mL centrifuge tubes and extracted overnight with 10 mL distilled water at 70°C. The homogenates were centrifuged at 12000 g for 20 min, and the supernatants were collected for the determination of soluble sugar, and the sediments were stored at −20°C for the starch content determination (Glassop et al., 2007). After the reaction of 1 mL of supernatants mixed with 1 mL of phenol and 5 mL of sulfuric acid for half an hour, the soluble sugar content was determined by using an ultraviolet spectrophotometer at 485 nm. The collected sediments were boiled with deionized water, and the supernatants were used for the starch content determination according to the method of DuBois et al. (1956). The NSC values were calculated as the sum of soluble sugar and starch content in different age groups of both non-flowering and flowering bamboo culms. Each determination was repeated three times.

#### Activity determination of starch-metabolizing enzymes

To determine the activities of starch-metabolizing enzymes, the crude enzyme solutions were extracted as described by Nakamura et al. (1989) and Sergeeva et al. (2012). The specimens (0.5 g) were thoroughly ground in liquid nitrogen and transferred to centrifuge tubes containing 1 mL of the extraction buffer, which comprised 100 mM HEPES/NaOH (pH 7.4), 5 mM MgCl_2_, 2 mM ethylenediaminetetraacetic acid (EDTA), 10% (v/v) glycerol, 0.1% BSA, 5 mM 1,4-ditjiothreitol (DTT) and 2% (w/v) polyvinyl pyrrolidone (PVP). The tubes were centrifuged at 12000 g at 4°C for 30 min. The supernatants were stored at 4°C for the determination of AGPase and soluble starch synthase (SSS) activities. The sediments were resuspended with 1 mL of extraction buffer for the activity determination of granular-bound starch synthase (GBSS). Each determination was repeated three times.

The determination of AGPase activities in non-flowering and flowering culm samples of different ages was carried out in centrifuge tubes, which comprised 100 mM HEPES/NaOH (pH 7.4), 3 mM PPi, 5 mM MgCl_2_, 4 mM DTT, 1.2 mM ADPG and 100 μL of crude enzyme solutions (Nakamura et al., 1989). Reactions were proceeded at 37°C for 30 min and then were boiled at 100°C for 10 min to terminate the reaction. After centrifugation at 12000 g for 10 min, 500 μL of the supernatants were mixed with 15 μL of 10 mM NAD and 1 μL of P-glucomutase (0.4 U) and glucose-6-phosphate dehydrogenase (0.35 U). The activities were calculated in terms of the increase of NADH in absorbance at 340 nm. The activities were calculated in µmol NADH per min per g fresh tissue.

The activities of SSS and GBSS were determined according to the methods of Nakamura et al. (1989). The reactions were conducted in 2 mL centrifuge tubes with 20 μL of crude enzyme solutions and 280 μL of reaction buffer, which comprised 50 mM HEPES/NaOH (pH 7.4), 0.7 mg amylopectin, 15 mM DTT, 1.6 mM ADPG, and enzyme preparation. The reactions were incubated at 37°C for 30 min and then were terminated at 100°C for 10 min after adding 100 μL of the reaction buffer, which included 50 mM HEPES/NaOH (pH 7.4), 200 mM KCl, 10 mM MgCl_2_, 4 mM PEP, and 1.2 U pyruvate kinase. After centrifugation at 12000 g for 10 min, 300 μL of the supernatants were mixed with 50 mM HEPES/NaOH (pH 7.4) buffer, 20 mM MgCl_2_, 10 mM glucose, and 2 mM NAD. The activities were measured as the increase in absorbance at 340 nm after the addition of 1 μL of hexokinase (1.4 U) and glucose-6-phosphate dehydrogenase (0.35 U). The activities of GBSS were determined by the same method, and the suspended pellet was mixed with the reaction buffer instead of the supernatants. The activities were also calculated in µmol NADH per min per g fresh tissue.

The determination of STP activities was carried out in a final volume of 1 mL solution, containing 50 mM HEPES/NaOH (pH 7.0), 10 mM Na_3_PO_4_, 0.4% soluble starch, 15 mM glucose-1,6-bisphosphate, 0.4 mM NAD, 1 U glucose-6-phosphate dehydrogenase, 1 U phosphoglucomutase, and 50 μL of crude enzyme solutions, according to the method of Appeldoorn et al. (1999). The reactions were proceeded at 37°C for 30 min and terminated by boiling for 10 min. The activities were measured by the increase in absorbance at 340 nm, and the activity was expressed as µmol NADH per min per g fresh tissue.

#### Activity determination of sucrose-metabolizing enzymes

The crude enzymes were extracted according to Moscatello et al. (2011). The specimens (0.5 g) were ground in a pre-cold mortar and transferred to centrifuge tubes containing 3 mL of the extraction buffer, which comprised 50 mM HEPES/NaOH (pH 7.5), 7.5 mM MgCl_2_, 1 mM EDTA, 2% PEG4000, 2% PVP and 5 mM DTT. The solutions were centrifuged at 3760 g at 4°C for 10 min (Yu et al., 2021). The supernatants were diluted to 3 mL, and were stored at 4°C for the determination of SAI and SUSY activities. Each determination was repeated three times. The activities of soluble acid invertase (SAI) and insoluble acid invertase (CWI) were determined according to Wang et al. (2019). Sucrose synthase (SUSY) activities were assayed according to Sung et al. (1994).

#### Statistical analysis

The presented data was the mean of three independent experiments. One-way ANOVA was used to compare the indicators obtained from culms of all ages and the levels of significance were determined by using the least significant difference (LSD) test. For the comparison of the indicators obtained from the flowering and non-flowering bamboo culms, an independent sample T-test was employed by using the SPSS 21.0 software (SPSS, Inc., Chicago, IL). Differences were considered significant at p< 0.05.

## Results

### Differences in culm diameter of non-flowering and flowering *B. tuldoides culms*

**Figure 1:**
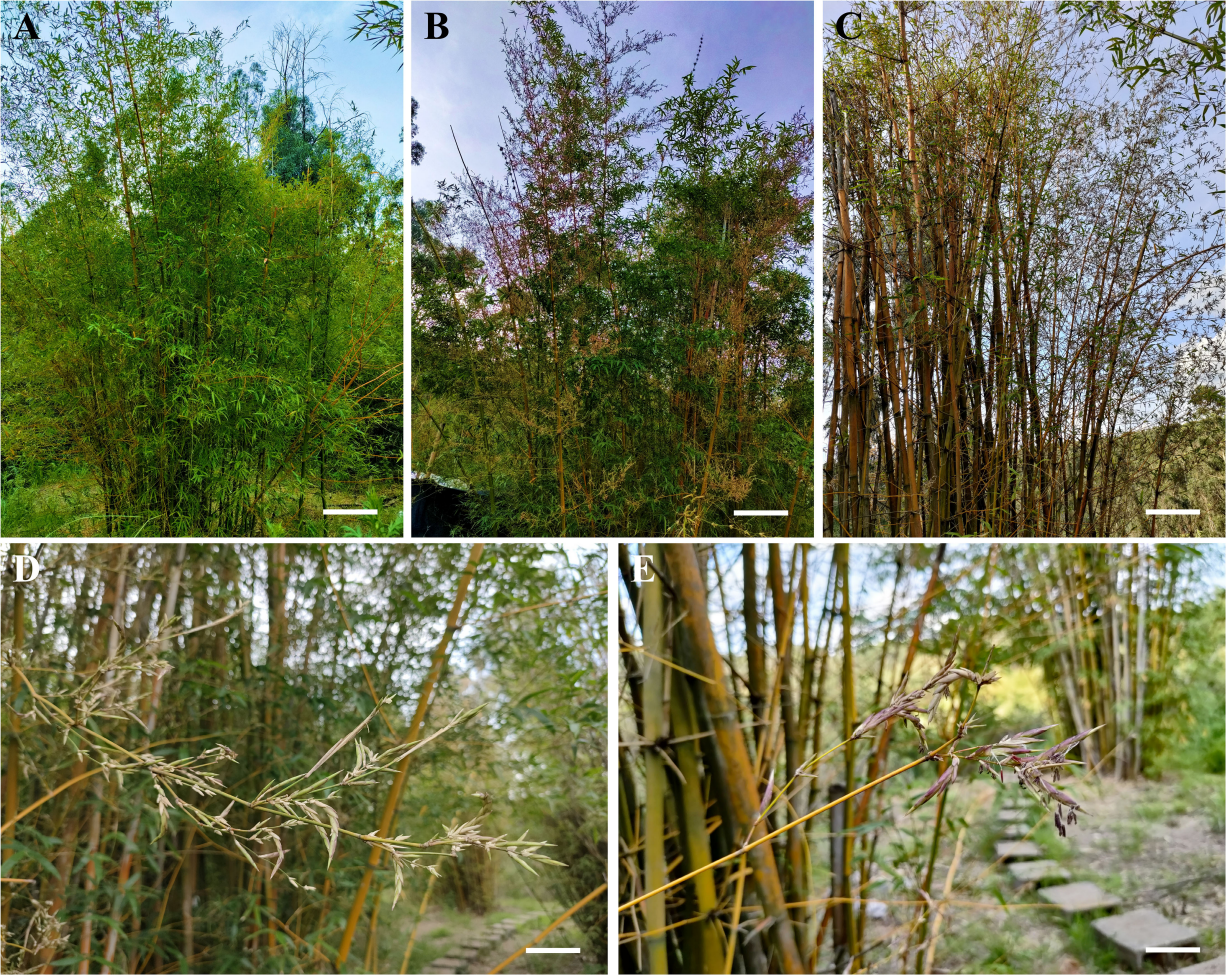
The difference in the growth of non-flowering and flowering *B. tuldoides* culms. **(A)** Non-flowering *B. tuldoides* culms. Bar=30cm. **(B, C)** Flowering *B. tuldoides* culms. Bar=20cm. **(D, E)** Morphology and growth of spikelets. Bar=3cm.

The field observations on the non-flowering and flowering *B. tuldoides* clusters showed that the non-flowering bamboo had green culms, luxuriant branches and leaves (**Figure 1A**). However, the flowering culms turned yellow, and a large number of leaves also turned yellow and dropped off (**Figures 1B, C**). New pale purple spikelets generated continuously on the branches of the flowering culms (**Figures 1D, E**). The flowering bamboo had bloomed for two years as the culm samples were gathered. Only the 3-year-old culms germinated before blooming, while the 3-month-old, 1-year-old and 2-year-old culms germinated during the blooming period.

**Figure 2:**
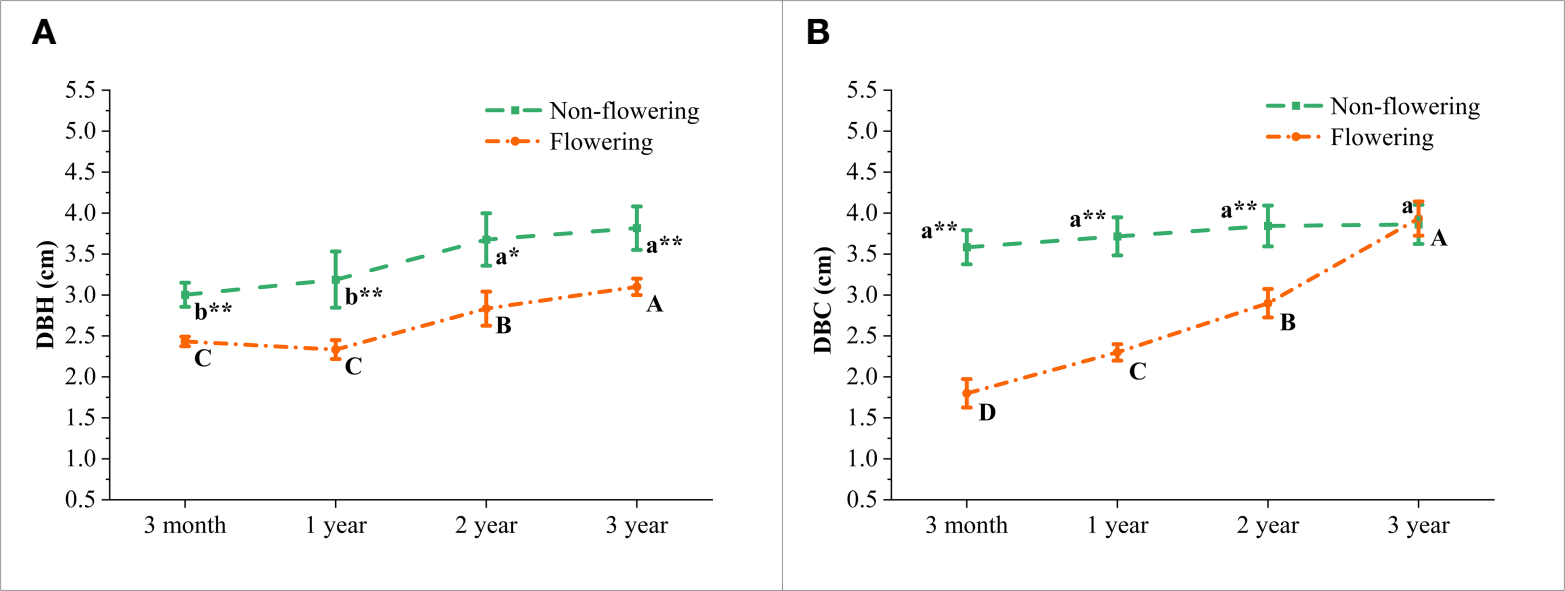
Morphology in the non-flowering and flowering *B.* *tuldoides* culms of different ages. **(A)** Changes of the diameter at breast height (DBH) of culms. **(B)** Changes of the diameter at culm bottom (DBC) of culms. Data were presented as mean ± SD (n=3). Different lowercase letters indicated significant differences with age in the non-flowering culms, and different uppercase indicated differences with age in the flowering culms at the level of p<0.05. * indicated significant difference at the level of p<0.05, and ** indicated significant difference at the level of p<0.01 between the non-flowering and flowering culms.

According to the wild observations, the diameters at breast height (DBH) and at culm bottom (DBC) of *B. tuldoides* showed a significant difference between the flowering and non-flowering clusters (**Figure 2**). It could be noticed that both DBH and DBC showed an upward trend with age with the highest values at the 3-year-old culms. This implied that flowering and non-flowering bamboo clusters might had an apparent degradation tendency, but this degradation was more significant in the flowering bamboo clusters. Moreover, there was no significant difference in the DBC of 3-year-old flowering and non-flowering culms (**Figure 2B**), which indicated that flowering had great negative impacts on the growth and development of culms. Therefore, it might be due to the huge nutrition consumption caused by the bamboo flowering that the size of new germinated culms become smaller and smaller.

### Vascular bundle morphology of non-flowering and flowering *B. tuldoides culms*

**Figure 3:**
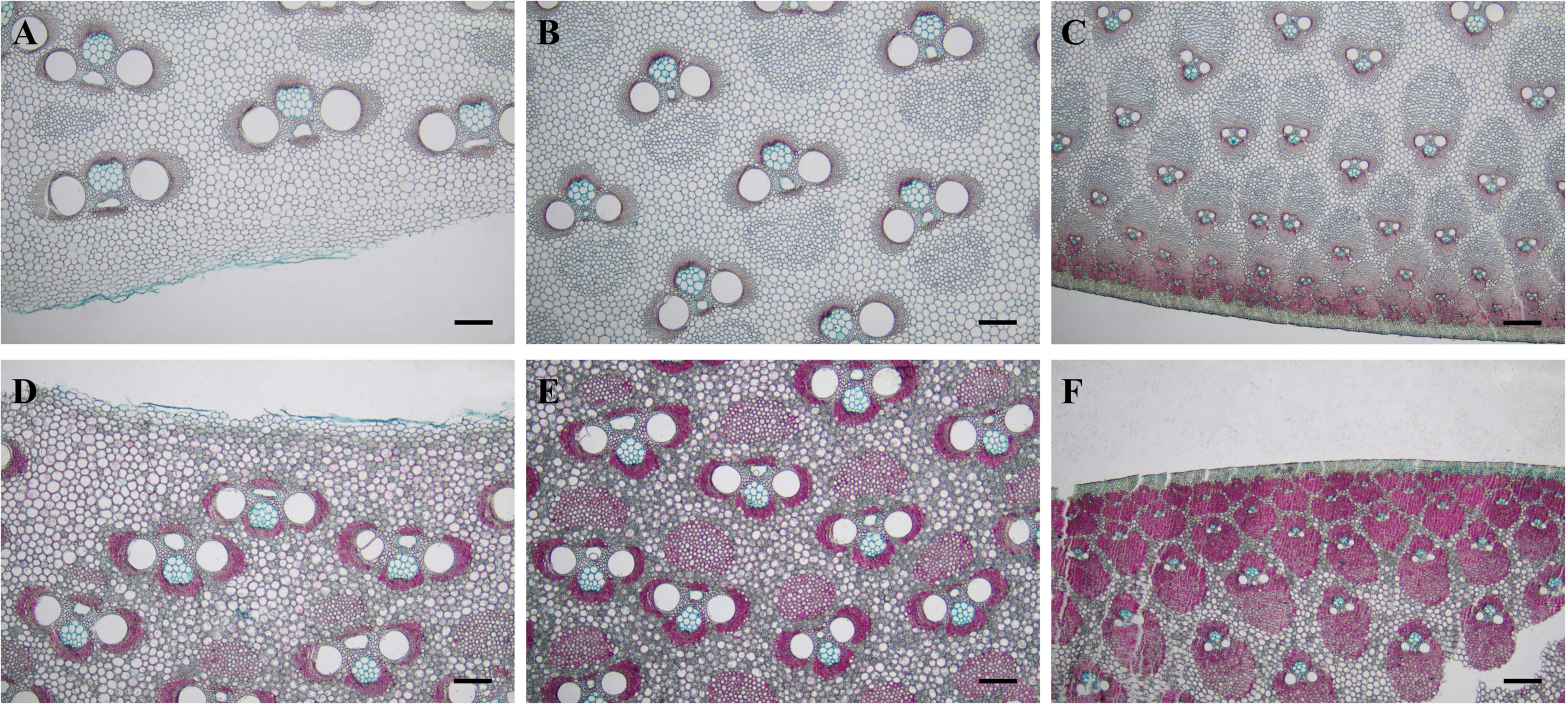
Vascular bundle morphology of *B. tuldoides* culms after the staining with safranin O and alcian blue. Each internode sample was cut into 15 cross-sections continuously for the observations. **(A–C)** Inner, middle, and outer zones in the 3-month-old culms. Bar=200 μm. **(D–F)** Inner, middle, and outer zones in the 1-year-old culms. Bar=200 μm.

Liese (1985) described five basic types of vascular bundles in different bamboo species, i.e., type I (open type), type II (slender waist type), type III (broken type), type IV (double-broken type), and type V (semi-open type). The middle part of 3-month and 1-year-old *B. tuldoides* culms showed three types of vascular bundles from the inner zone to the outer zone in transverse sections, i.e., open type (**Figures 3A, D**), broken type (**Figures 3B, E**) and semi-differentiation type (**Figures 3C, F**).

In the inner zone of culms, the vascular bundles showed no isolated fiber sheath, and were more closed to the open-type in morphological structure (**Figures 3A, D**). While in the middle zone, the vascular bundles were stable in shape, which were consisted of two parts, i.e., the central vascular bundle and the isolated fiber sheath. The isolated sheath was localized at the place close to the protoxylem and larger than the fiber sheaths of the xylem and phloem in size (**Figures 3B, E**), and this kind of vascular bundle belonged to the broken type. The vascular bundles were densely distributed at the outer zone with smaller size and with no isolated fiber sheath, which could be identified as the semi-differentiation type (**Figures 3C, F**).

### Changes in fiber morphology of non-flowering and flowering *B. tuldoides culms*

**Figure 4:**
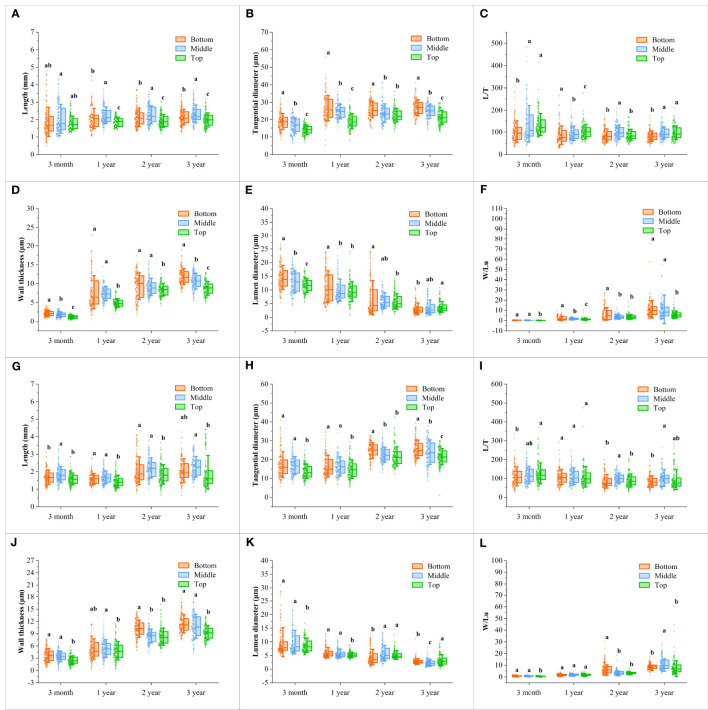
Changes of the fiber characteristics in the *B. tuldoides* culms with portions and age. Each determination was repeated three times and a total of 50 fibers were measured each time. **(A–F)** Changes of the fiber characteristics in the non-flowering culms with portions and age. **(A)** Length. **(B)** Tangential diameter. **(C)** L/T. **(D)** Wall thickness. **(E)** Lumen diameter. **(F)** W/Lu. **(G–L)** Changes of the fiber characteristics in the flowering culms with portions and age. **(G)** Length. **(H)** Tangential diameter. **(I)** L/T. **(J)** Wall thickness. **(K)** Lumen diameter. **(L)** W/Lu. Different lowercase letters indicated significant differences of different portions in the same age at the level of p<0.05.

**Figure 5:**
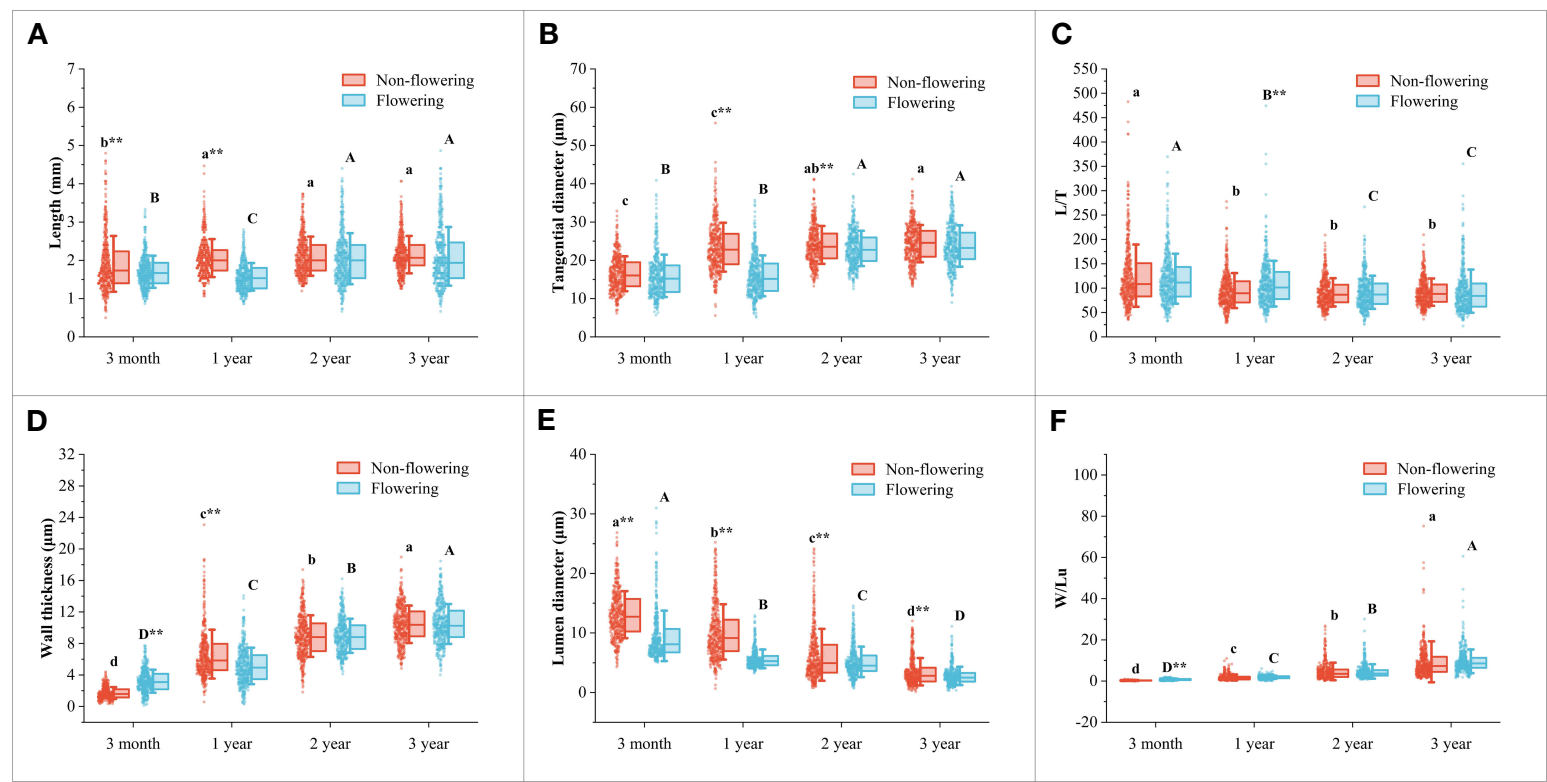
Changes of the fiber characteristics in the non-flowering and flowering *B. tuldoides* culms with age. Each determination was repeated three times and a total of 150 fibers were measured each time. **(A)** Length. **(B)** Tangential diameter. **(C)** L/T. **(D)** Wall thickness. **(E)** Lumen diameter. **(F)** W/Lu. Different lowercase letters indicated significant differences with age in the non-flowering culms and different uppercase indicated differences with age in the flowering culms at the level of p<0.05. ** indicated significant difference at the level of p<0.01 between the non-flowering and flowering culms.

The fiber morphological characteristics were compared between the flowering and non-flowering culms of different ages, which mainly included fiber length, tangential diameter, slenderness ratio, wall thickness, lumen diameter, and Runkel ratio (**Figures 4**, **5**).

It could be noticed that the fiber length (**Figures 4A, G**) and tangential diameter (**Figures 4B, H**) in both flowering and non-flowering culms gradually increased with age, especially in the first growth season, both of which significantly increased, and a similar trend was also observed in the wall thickness (**Figures 4D, J**) and W/Lu (**Figures 4F, L**), while the lumen diameter (**Figures 4E, K**) showed an opposite trend. The L/T did not show an apparent trend (**Figures 4C, I**). These results indicated that the fibers basically completed their elongation in the first growth season, and then continuously deposited their walls.

Fiber morphology also showed significant differences and the same variation trend with height in both flowering culms and non-flowering culms (**Figures 4A–L**). The fiber length showed the highest values in the middle parts but the shortest values in the top parts (**Figures 4A, G**). A similar trend was also shown in L/T with the highest values in the middle parts (**Figures 4C, I**). However, the fiber tangential diameter and wall thickness of fibers decreased constantly with height and had the highest values at the bottom (**Figures 4B, H**; **Figures 4D, J**). Moreover, the W/Lu also showed larger values in the bottom parts (**Figures 4F, L**).

Generally, the fiber morphological characteristics showed similar variation trends in both flowering and non-flowering culms. However, the fiber length (**Figure 5A**), tangential diameter (**Figure 5B**), wall thickness (**Figure 5D**) and lumen diameter (**Figure 5E**) decreased significantly in the flowering culms of 3-month, 1-year-old as compared to those of non-flowering culms. Meanwhile, the tangential diameter and lumen diameter of 2-year culms were also lower in the flowering clusters than in the non-flowering clusters. There was no significant difference in fibers between the flowering and non-flowering culms of 3 years old. This coincided with the fact that the 3-year-old culms had completed their height growth before entering into the flowering period.

### Moisture content and chemical composition of non-flowering and flowering *B. tuldoides culms*

**Figure 6:**
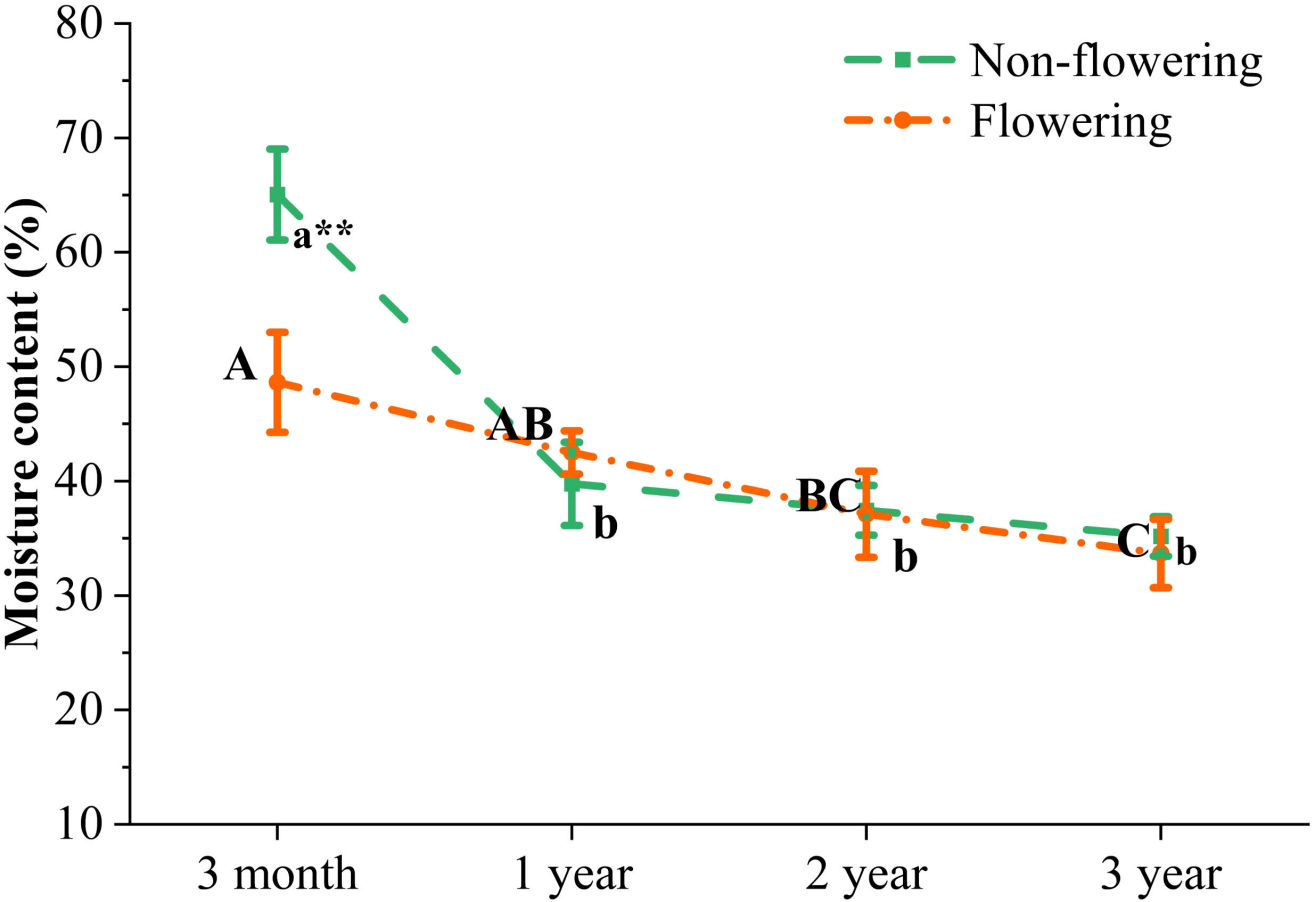
Changes of moisture content in the non-flowering and flowering *B.* *tuldoides* culms of different ages. Data were presented as mean ± SD (n=3). Different lowercase letters indicated significant differences with age in the non-flowering culms, and different uppercase indicated differences with age in the flowering culms at the level of p<0.05. * indicated significant difference at the level of p<0.05, and ** indicated significant difference at the level of p<0.01 between the non-flowering and flowering culms.

Moisture played a vital role in the growth of bamboo plants. The moisture content of *B. tuldoides* culms decreased significantly from 3 months to 1 year, and also showed a constantly decreasing trend in the following ages, but the difference was not significant. After flowering, the moisture content decreased significantly in the 3-month-old bamboo culms, but did not show significant difference between the flowering and non-flowering culms of 1, 2 and 3 years (**Figure 6**). Therefore, the flowering influenced more significantly the moisture content of young culms.

**Figure 7:**
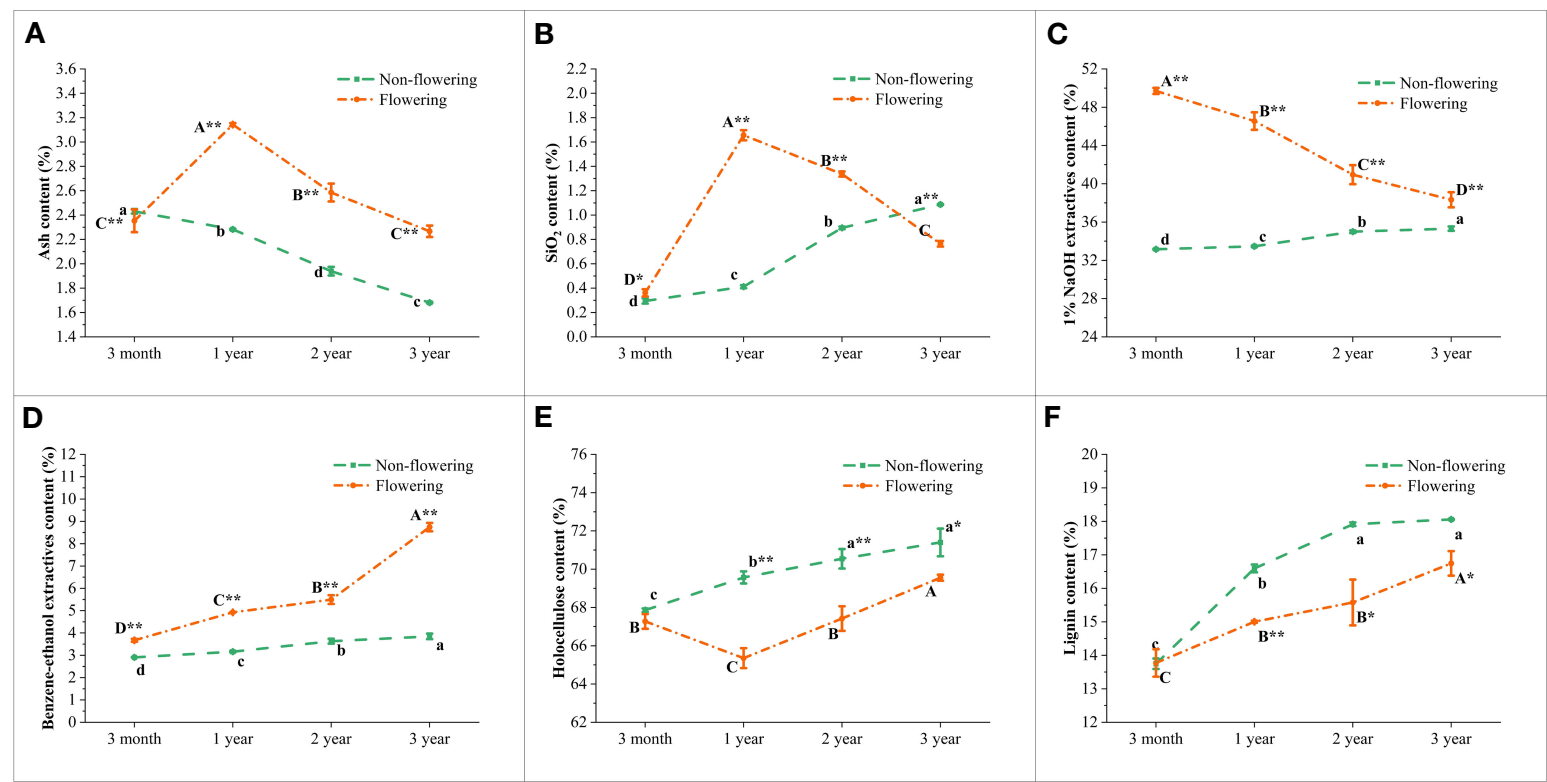
Changes of the major chemical components in the non-flowering and flowering *B.* *tuldoides* culms of different ages. Data were presented as mean ± SD (n=3). **(A)** Ash content. **(B)** SiO_2_ content. **(C)** 1% NaOH extractives content. **(D)** Benzene-ethanol extractives content **(E)** Holocellulose content. **(F)** Lignin content. Different lowercase letters indicated significant differences with ages in the non-flowering culms and different uppercase indicated differences with ages in the flowering culms at the level of p<0.05. * indicated significant difference at the level of p<0.05, and ** indicated significant difference at the level of p<0.01 between the non-flowering and flowering culms.

The contents of chemical components in the flowering and non-flowering *B. tuldoides* culms were measured and compared (**Figure 7**). In the non-flowering bamboo, the ash content decreased significantly with age (**Figure 7A**), while the SiO_2_ content showed the opposite trend (**Figure 7B**). After flowering, both ash and SiO_2_ contents increased significantly and then decreased significantly with age with the highest contents in the 1-year-old culms.

It could be noticed that the 1% NaOH and benzene-ethanol extractives (**Figures 7C, D**) and lignin contents (**Figure 7F**) showed a significantly increasing trend with age in the non-flowering culms. The holocellulose contents also increased significantly in the non-flowering culms from 3 months to 1 year, but increased slightly in the following years. A similar trend was also observed in the contents of benzene-ethanol extractives and lignin in the flowering bamboos (**Figures 7D, F**), but 1% NaOH extractives content decreased significantly and constantly with age (**Figure 7C**). The holocellulose content of the flowering bamboos decreased first and then increased constantly in the following years (**Figure 7E**), which indicated that the ability of cell wall synthesis of the young *B. tuldoides* culms might be inhibited after flowering.

In general, the chemical components showed a similar variation trend in the flowering and non-flowering culms with age except the contents of SiO_2_ and 1% NaOH extractives. Meanwhile, the holocellulose and lignin contents were lower in the flowering culms as compared to the non-flowering culms, while the other chemical components showed higher contents in the flowering culms.

### Endogenous soluble sugar, starch and NSC contents of non-flowering and flowering *B. tuldoides culms*

**Figure 8:**
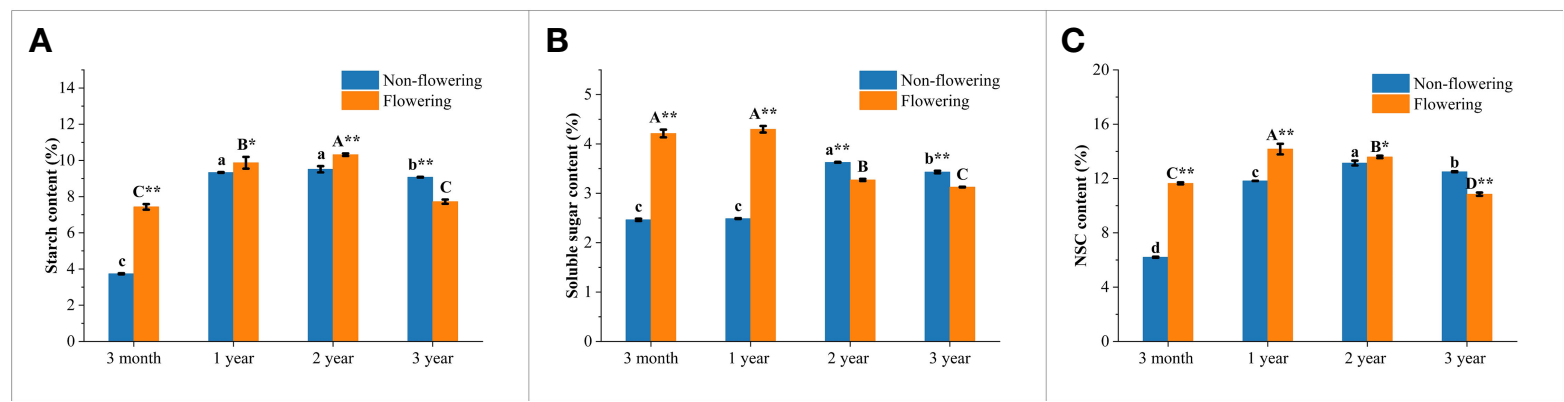
Changes of the carbohydrate storage in the non-flowering and flowering *B.* *tuldoides* culms of different ages. Data were presented as mean ± SD (n=3). **(A)** Starch content. **(B)** Soluble sugar content. **(C)** NSC content. Different lowercase letters indicated significant differences with age in the non-flowering culms and different uppercase indicated differences with age in the flowering culms at the level of p<0.05. * indicated significant difference at the level of p<0.05, and ** indicated significant difference at the level of p<0.01 between the non-flowering and flowering culms.

**Figure 9:**
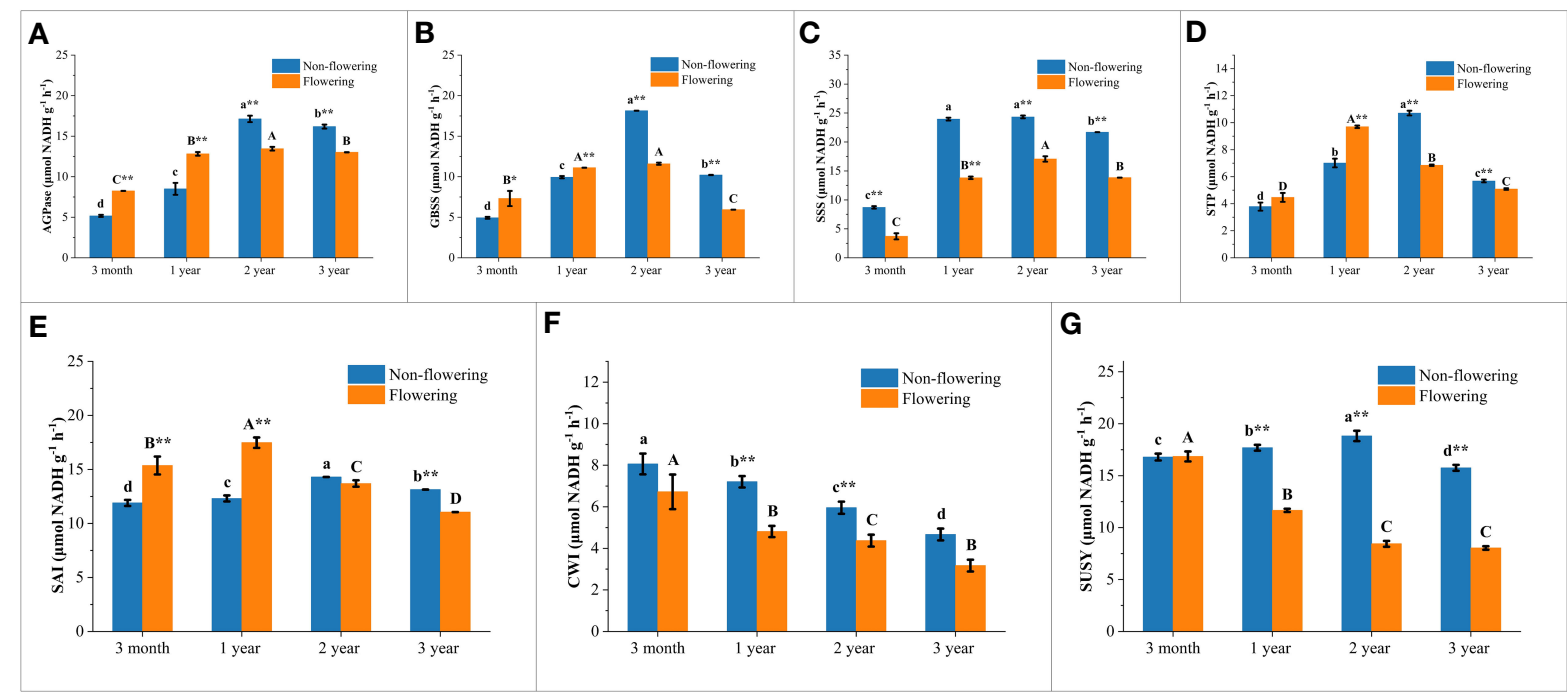
Changes of sugar-metabolizing enzymatic activities in the non-flowering and flowering *B.* *tuldoides* culms of different ages. Data were presented as mean ± SD (n=3). **(A)** AGPase activities. **(B)** SSS activities. **(C)** GBSS activities. **(D)** STP activities. **(E)** SAI activities. **(F)** CWI activities. **(G)** SUSY activities. Different lowercase letters indicated significant differences with age in the non-flowering culms and different uppercase indicated differences with age in the flowering culms at the level of p<0.05. * indicated significant difference at the level of p<0.05, and ** indicated significant difference at the level of p<0.01 between the non-flowering and flowering culms.

The contents of soluble sugar, starch and NSC in the non-flowering and flowering *B. tuldoides* culms were measured and compared (**Figures 8A–C**). The activities of sugar-metabolizing enzymes were also measured and compared (**Figures 9A–G**), so as to analyze the influences of flowering on sugar metabolism during bamboo culm growth and development.

It could be noticed that the starch content (**Figure 8A**) increased constantly and significantly from 3-month to 2-year-old culms and then decreased in both flowering and non-flowering bamboo clusters. The flowering culms showed higher starch content than the non-flowering culms at the age of 3 months, 1 and 2 years, but lower values at the age of 3 years. However, the flowering culms showed significantly lower starch content than the non-flowering culms at age of 3 years, which implied that the 3-year-old culms supplied carbohydrates for the 3-month and 1- and 2-year-old culms in the flowering clusters.

In the non-flowering bamboo, the soluble sugar content (**Figure 8B**) increased constantly from 3-month culms to 2-year-old culms, and then decreased significantly in the 3-year-old culms. While in the flowering culms, the soluble sugar content slightly increased from 3 months to 1 year, and then constantly decreased in the following year. It was also noticed that the soluble sugar content was significantly higher in the 3-month and 1-year-old flowering culms but was significantly lower in the 2- and 3-year-old flowering culms as compared to the non-flowering culms. These results implied that the soluble sugar was mainly consumed in the young flowering culms.

The NSC content (**Figure 8C**) showed the same trend as starch content in the non-flowering bamboo with the highest NSC content in the 2-year-old bamboo culms. In the flowering bamboo, the NSC content increased significantly from 3 months to 1 year, and then decreased in the following years. Additionally, the NSC content was significantly higher in the flowering culms than the non-flowering culms at the age of 3 months, 1 and 2 years, while the 3-year-old culms showed lower content than the non-flowering culms. These results indicated that bamboos required a large amount of carbohydrates during the flowering period, and the 3-year-old culms provided a large amount of energy for the flowering culms.

### Localization of starch grains in the flowering and non-flowering *B. tuldoides culms*

**Figure 10:**
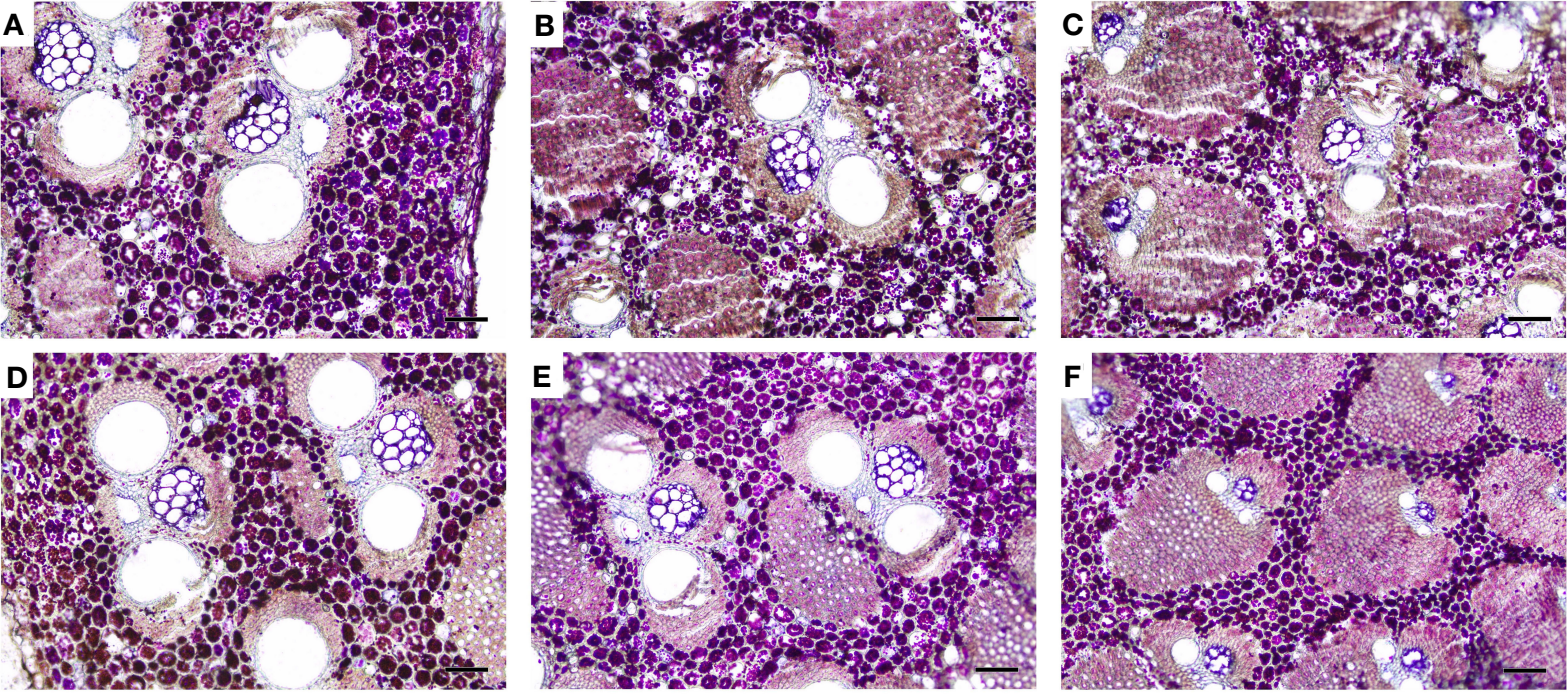
Distribution of starch grains in the upper part (internode 13) of 2-year-old *B. tuldoides* culms after the staining of PAS reaction. Each sample was cut into 15 cross-sections continuously for the observations. **(A–C)** Inner, middle, and outer zones of the non-flowering culms. Bar=200μm. **(D–F)** Inner, middle, and outer zones of the flowering culms. Bar=200μm.

The distribution of starch grains was analyzed in the top part of 2-year-old culms, which was mainly due to the fact that the 2-year-old culms showed the highest starch contents in both flowering and non-flowering bamboo clusters (**Figures 8A**, **10**). According to the PAS reaction, a large number of starch grains were observed and mainly localized in the parenchyma cells between vascular bundles in both flowering and non-flowering culms (**Figure 10**). It could also be noticed that there were more starch grains in the outer zone than the middle and inner zones in both flowering and non-flowering culms. Additionally, the 2-year-old flowering culms also showed more starch grains than the non-flowering culms, which coincided with the determination of starch contents (**Figure 8A**).

### Activities of starch-metabolizing enzymes of non-flowering and flowering *B. tuldoides culms*

In the synthesis direction of starch, the activities of AGPase, GBSS and SSS showed a variation trend that increased firstly and then decreased significantly with the highest values in the 2-year-old culms in both flowering and non-flowering bamboo clusters (**Figures 9A–C**). It could also be noticed that the AGPase and GBSS activities were significantly higher in the flowering bamboo culms than in the non-flowering bamboo culms at the age of 3 months and 1 year, but significantly lower at the age of 2 and 3 years (**Figures 9A, B**). However, the SSS activities were significantly decreased in the flowering culms at all age classes (**Figure 9C**). Generally, flowering significantly influenced the starch synthesis in bamboo culms.

The STP activities showed a similar trend that increased first and then decreased significantly with age with the highest values in the 2-year-old non-flowering culms but with the highest values in the 1-year-old flowering culms (**Figure 9D**). Meanwhile, the STP activities were significantly higher in the flowering bamboos of 3 months and 1 year but lower in the flowering culms of 2 and 3 years old as compared to the non-flowering bamboos. This variation trend was completely consistent with that of AGPase and GBSS (**Figures 9A, B**). This also implied that flowering could improve the capacity of starch synthesis and degradation in the young bamboo culms, but decreased their activities in the mature bamboos.

### Activities of sucrose catabolizing enzymes of non-flowering and flowering *B. tuldoides culms*

The activities of sucrose-catabolizing enzymes in culms, such as SAI, CWI, and SUSY, were determined in the non-flowering and flowering bamboos at different age classes (**Figures 9E–G**). Like STP, the SAI activities also increased firstly and then decreased constantly with age with the highest values in 1-year-old culms in both flowering and non-flowering clusters (**Figure 9E**). This revealed that the SAI activities increased in young culms but decreased in mature and old culms after flowering.

As for CWI, the activities showed a continuously decreasing trend with age in both flowering and non-flowering bamboo clusters (**Figure 9F**). Meanwhile, the activities were decreased in the flowering culms of all age classes as compared to the non-flowering culms, which implied that flowering could apparently decrease the CWI activities.

The metabolic activities catalyzed by SUSY were related to the biosynthesis process of cell walls (Winter and Huber, 2000). In the non-flowering culms, SUSY activities exhibited a similar trend to that of SAI, which increased first and then decreased with age with the highest values in the 2-year culms (**Figures 9E, G**). However, the SUSY activities in the flowering culms decreased constantly with age and were far lower than those in the non-flowering culms of all age classes, except for the culms of 3 months. This indicated that the cell wall synthesis might be significantly inhibited after flowering.

### Correlation analysis

**Figure 11:**
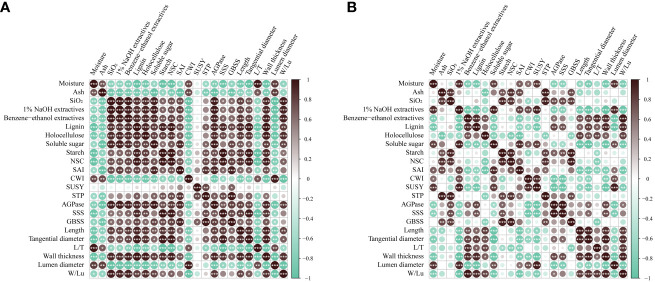
Correlation analysis of physiological indexes in *B. tuldoides* of different ages. **(A)** Correlation analysis of physiological indexes in the non-flowering culms. **(B)** Correlation analysis of physiological indexes in the flowering culms. The darkness of the color indicated the ranking: the black circle marked the value of positive correlation, and the green circle marked the value of negative correlation. * indicated significant correlation at 0.05 level, ** indicated significant correlation at 0.01 level, and *** indicated significant correlation at 0.001 level.

In order to further reveal the influences of flowering on the chemical properties of *B. tuldoides* culms and carbohydrate metabolism, a correlation analysis between various indicators was conducted (**Figure 11**).

For the non-flowering *B. tuldoides* culms, the results showed that almost all chemical components and the length, tangential diameter, wall thickness of fibers showed significant and positive correlations with soluble sugar, starch, NSC contents and the activities of SAI, STP, AGPase, and GBSS, except ash and moisture contents (**Figure 11A**). This indicated that the accumulation of chemical components and the elongation of fibers were closely related to the sugar metabolism during the growth and development of *B. tuldoides* culms.

After flowering, 1% NaOH extractives correlated significantly and positively with the soluble sugar content and the activities of sucrose-catabolizing enzymes (**Figure 11B**), while the length, tangential diameter, wall thickness of fibers, and the contents of benzene-ethanol extractives, lignin and holocellulose showed negative correlations with the contents of soluble sugar, starch and NSC and the activities of sucrose catabolizing enzymes. These results might be mainly due to the fact that most carbohydrates stored in the culms were consumed for the flowering, not for the elongation and maturation of fibers and chemical components accumulation in the flowering culms, which further decreased their correlations.

## Discussion

Bamboos rarely bloom and remain in a vegetative stage for decades or even a century, followed by flowering and death (Zheng et al., 2020). With the progress of flowering, the nutrients in organs such as leaves, stems, and roots gradually decreased (Gao et al., 2002; Zhan and Li, 2007). Plant flowering was considered as an aging phenomenon in which the nutrient consumption of plants increased while their ability to synthesize energy decreased (Ge et al., 2017). As a result, vegetative growth was inhibited, leading to the phenomenon of “starvation death” and finally resulting in a large number of deaths in bamboo stands after flowering (Chai et al., 2006). Marchesini et al. (2009) found that a large-scale flowering event occurred throughout the entire bamboo stand in *Chusquea culeau*, followed by withering and death, resulting in a mortality rate of 96.5%. With the passage of time, the biomass of dead matter decreased significantly. However, few researches have focused on the bamboo timber characteristics, physiological and biochemical aspects of bamboo after flowering.

### Morphological observations of non-flowering and flowering *B. tuldoides culms*

After flowering, many spikelets appeared on the branches, and a large of leaves turned yellow and fell off. Then the bamboo culms turned yellow, and this might be due to the fact that the decrease in the number of leaves weakened the photosynthetic capacity, and flowering also required a large amount of carbohydrates, resulting in the inability of the bamboo culm to perform normal physiological activities. This might also be one of the main reasons why the flowering culms became yellow as compared to the non-flowering culms.

During the normal growth and development of bamboo, there was basically no significant change in the DBC of shoots and mature culms (Wang, 2017). Therefore, there was no difference in the DBC of bamboo culms at 3 months, 1 year, 2 years and 3 years in the non-flowering culms. However, the DBC of those flowering culms increased with age. It was well known that a large amount of energy would be consumed during flowering, which limited the growth of new culms. Hence, the newly germinated culms became thinner and thinner due to the insufficient energy supply.

### Anatomical structure changes of non-flowering and flowering *B. tuldoides culms*

The shape and size of vascular bundles varied with culm zones. The vascular bundles of *B. tuldoides* in the inner zone were shorter and larger, while those in the outer zone were longer and smaller and more densely distributed, which made the density and mechanical strength at the outer zone higher than the inner (Zhou, 1981; Liese, 1985). After completing high growth, the fiber cells and parenchyma cells gradually lignified from the outer zone to the inner zone, which was consistent with the conclusion of Itoh and Shimaji (1981).

The fiber length of *B. tuldoides* increased significantly within 1 year and slightly increased in the following years. The tangential diameter also showed a similar rule. This was because bamboos usually reached their maturity in one year, which was also reported by Xiang et al. (2020). As we all know that most bamboo plants usually completed their growth within 1 to 2 years without secondary growth and various cells differentiated and matured from bottom to top (Wang et al., 2011). Once the height growth of bamboo culms was completed, the fibers would stop increasing in length and tangential diameter, but the wall thickness could continue to deposit and form a polylamellate structure with age (Wang et al., 2011), and constantly thickened within the following years (Gan and Ding, 2006). The fibers of *B. tuldoides* also showed the similar trend in that the fiber wall thickness and W/Lu ratio increased significantly. The tangential diameter was the sum of the lumen diameter and double wall thickness, the significant increase of fiber wall thickness led to a significant decrease in the lumen diameter. Other researchers reported that the bamboo could still deposit their fiber walls even at the age of 12 years old (Liese and Weiner, 1996; Murphy and Alvin, 1997).

The fiber morphology also showed significant differences with height with the highest length in the middle part and the largest tangential diameter in the bottom. Similar trends were also observed in *Fargesia yunnanensis* (Wang et al., 2011) and *Dendrocalamus giganteus* (Wang et al., 2016). Liese (1998) believed that the length of the fibers was related to the internode length. The fiber wall thickness and W/Lu ratio at the top of *B. tuldoides* were the smallest, which was consistent with the results of Liese (1998) and Wang et al. (2016). Wang et al. (2011) also reported that the bottom showed the largest wall thickness. This phenomenon might be due to the fact that the fiber cells at the bottom were more huge than those at the top, which had higher spaces for cell wall deposition. The lumen diameter decreased with age and height, which might be mainly because the fibers in the upper part had a smaller tangential diameter as compared to those in the bottom, and the wall thickness also increased constantly with age.

The longer fibers had higher tear and tensile strength (Zhan et al., 2016). The fiber tangential diameter was closely related to the cross area of the fibers, and the wider fiber had a larger cross area, which was beneficial for producing high-quality and high-strength paper (Anupam et al., 2016). After flowering, the fiber length and tangential diameter of 3-month and 1-year culms were significantly lower than those of non-flowering culms, indicating that flowering affected the growth and development of fibers. This was also consistent with the significant decrease in DBH and DBC of bamboo culms after flowering. The bamboo stands of *B. tuldoides* had been in flowering period for two years, and most leaves fell off and significantly decreased their photosynthesis. Moreover, the flowering consumed a large amount of carbohydrates, which further limited the young culm growth and resulted in a significant decrease in the fiber length and tangential diameter. However, the culms of 2 and 3 years had completed their cell elongation before flowering, and hence no significant difference occurred in fiber morphology.

### Chemical properties of non-flowering and flowering *B. tuldoides culms*

The moisture content in *B. tuldoides* culms decreased gradually with age. Wang et al. (2009) found that the moisture content of *F. yunnanensis* culms also decreased with age. Zhan et al. (2015) reported the same rule in *Fargesia fungosa* culms. Hisham et al. (2006) found that the *Gigantochloa scortechinii* culms at the highest age class (6.5 years) showed the lowest moisture content. Zhan et al. (2015) believed that the decrease in moisture content with age might be related to the lignification in vascular bundles and parenchyma cells. However, the decrease in moisture content in the flowering bamboo culms might be related to the decrease in the water absorption capacity after flowering.

The ability of bamboo to resist adversity was closely related to its chemical compositions (Mohmod, 1993). The ash content of non-flowering *B. tuldoides* decreased significantly with age, which was consistent with the results of Zhang et al. (2002) reported in moso bamboos (*Phyllostachys edulis*). Zhan et al. (2015) also found a similar trend in *F. fungosa*. The decrease in ash content might be related to the decrease in the absorption capacity of inorganic salts from soil with age. On the contrary, the SiO_2_ content constantly increased with age in the form of phytolith in bamboo to resist the invasion of insects. However, higher ash and SiO_2_ contents could adversely affect machining and alkali recovery during processing.

Benzene-ethanol extracting solution could dissolve wax, fat, oil, and a small amount of gum, while 1% NaOH solution could dissolve low-molecular-weight hemicellulose and part of lignin (Yuan et al., 2010). The degree of fungal decay or degradation could be expressed by the solubility of wood in 1% NaOH solution (Maulana et al., 2020). During the bonding process of adhesive products, such as strand board and plywood, high extractives contents could inhibit the penetration of the adhesive, resulting in low mechanical strength (Maloney, 1993). In the pulping process, the extracts in the form of wax and fat would reduce the bonding strength between the fibers, increase the consumption of alkali, and slow down the delignification (Maulana et al., 2020). Materials with low contents of 1% NaOH and benzene-ethanol extractives made it easier for chemicals to penetrate the material, which could usually be used to produce high-quality paper (Fatriasari and Hermiati, 2008). In our study, both benzene-ethanol extractives and 1% NaOH extractives increased with age in both flowering and non-flowering bamboo. The variation trend of benzene-ethanol extractives content was consistent with the results of Li et al. (2007); Wang et al. (2011) and Wang et al. (2016). The increase in 1% NaOH extractives content with age might be related to the accumulation of soluble sugar and starch in bamboo.

The materials with high holocellulose content could usually be used to produce high-quality paper (Afrifah et al., 2022), while those with high lignin contents would consume a large number of chemicals in the pulping process (Li et al., 2016). The contents of holocellulose and lignin increased constantly in *B. tuldoides* culms with age. This was mainly due to the constant secondary wall deposition and lignification in fibers and parenchyma cells (Wang et al., 2011). After the bamboos completed their height growth, the fiber walls also deposited continuously from the 1st year to the 3rd year, and the contents of holocellulose and lignin also increased gradually (Gritsch et al., 2004). Wang et al. (2011) and Wang et al. (2016) also reported in other bamboos that holocellulose content and lignin content increased with age.

The contents of ash, SiO_2_, 1% NaOH extractives, and benzene-ethanol extractives in *B. tuldoides* culms increased significantly after flowering at all ages. The 1-year-old flowering bamboos had the highest ash and SiO_2_ content, which might be due to the fact that bamboo required more nutrients during flowering, and mineral elements were the necessary nutrients for bamboo growth and metabolism. The bamboo culms absorbed a large number of mineral elements from the soil, which might be the main reason that a sharp increase occurred in ash and SiO_2_ contents in the flowering bamboo culms of all ages.

Cell walls were the main influencing factors for plant growth and development, moisture transport, and protective support (Keegstra, 2010). Cellulose, hemicellulose, and lignin were the main chemical components of bamboo culms (Liang et al., 2019), and their content changes could affect the structure and stability of fiber cell walls. The natural organic combination of the three chemical components determined the structure and properties of bamboo cell walls, and ultimately affected the processing and utilization of bamboo (Liu et al., 2016). It might be mainly because a large quantity of carbohydrates stored in bamboo culms were consumed during flowering, which further limited the cellulose synthesis. Hence, the flowering *B. tuldoides* culms showed lower contents of holocellulose and lignin as compared to those non-flowering culms at all age classes.

### Carbohydrate metabolism changes of non-flowering and flowering *B. tuldoides culms*

The endogenous soluble sugar and starch were the main forms of carbohydrates in plant vegetative tissues (Khalil et al., 2006). Soluble sugar was the substrate for starch synthesis, and when carbohydrate supply exceeded the demand, starch accumulated in tissues (Scofield et al., 2009). Carbohydrate metabolism could continuously provide energy for plant bodies, with STP involved in starch degradation in plants, and AGPase, SSS, and GBSS involved in starch synthesis (Wang et al., 2007). In the present works, all the activities of AGPase, SSS, and GBSS increased with age and began to decrease after 2 years. The starch content also showed a similar trend. This indicated that the starch synthesis began to decrease after two years in both flowering and non-flowering *B. tuldoides* culms.

SAI, CWI, and SUSY hydrolyzed soluble sugar, and their substrate shifted to glycolysis and cellulose synthesis pathways (Wang et al., 2007). SAI activities in rapidly growing tissues were usually higher and were involved in osmotic regulation, cell enlargement, and the sugar composition of sink tissues, while CWI was involved in phloem unloading and sucrose decomposition (Zhu et al., 1997; Roitsch and González, 2004). The metabolic activities catalyzed by SUSY were related to the biosynthesis process of cell walls (Winter and Huber, 2000). In the non-flowering culms of *B. tuldoides*, SAI and SUSY showed a similar trend that significantly increased trend at first, and then decreased significantly with age. This was related to the rapid growth and cellulose synthesis in young culms.

Plants usually consumed a large amount of energy during their flowering period. Many researchers had focused on exploring key genes involved in bamboo flowering, but the influences on the carbohydrate metabolism in bamboo culms were still unclear. In the present study, the soluble sugar and starch contents in the 3-month and 1-year-old flowering bamboo culms were significantly higher than those in non-flowering bamboo culms. Meanwhile, the activities of SAI and STP were also significantly increased. This indicated that the starch and sucrose catabolism was significantly increased in the flowering bamboo culms for their energy consumption.

When the supply exceeded the demand, the soluble sugar was converted into starch for storage. In the 2- and 3-year-old flowering culms, the activities of SSS and GBSS decreased significantly and the activities of SAI and SUSY also showed the same trend, but the starch and soluble sugar contents increased significantly as compared to the non-flowering culms. All these results revealed that most carbohydrates were transported to the young flowering culms for their energy consumption. In addition, the output of carbohydrates from the flowering mature culms also decreased their SUSY activities, which further decreased the cellulose synthesis of the flowering bamboo. Therefore, the holocellulose content was decreased in the flowering culms as compared to those non-flowering culms. This indicated that the flowering could affect the chemical components of the bamboos indeed.

Correlation analysis showed that the physiological metabolic processes of *B. tuldoides* accelerated during flowering, and increased the nutritional absorption capacity from the external environment and consumed a large number of carbohydrates, which resulted in an increase in extracts, ash, and SiO_2_ content. The correlation analysis also revealed that the accelerated carbohydrate catabolism in the flowering culms limited their cellulose, lignin contents and elongation of fibers. Therefore, the large-scale flowering could apparently cause declination in the quality of bamboo stands.

## Conclusion

The fiber morphology of *B. tuldoides* varied with age and height. The length, tangential diameter, wall thickness, and W/Lu ratio of fiber increased with age, but the lumen diameter decreased with age and height. In addition, the chemical composition of *B. tuldoides* culms also varied with age. With the increase of age class, the moisture and ash contents gradually decreased, while SiO_2_, 1% NaOH extractives, benzene-ethanol extractives, holocellulose, and lignin contents gradually increased. The contents of soluble sugar, starch and NSCs also increased continuously with the bamboo growth and development, and the sugar metabolism played an important role in the bamboo development. After flowering, *B. tuldoides* consumed a large amount of nutrients and the carbohydrate metabolism was accelerated, and then the fiber growth was limited. The fiber length and tangential diameter in the young culms became shorter as compared to those of the non-flowering culms. The holocellulose content also decreased in the flowering culms as compared to the non-flowering culms, which indicated that the flowering culms were not suitable for papermaking. Moreover, the increase of ash, SiO_2_ and extractives contents had a significant negative impact on the anatomical and chemical properties of culms and significantly decreased their utilization as raw materials for paper making and others.

## Data availability statement

The raw data supporting the conclusions of this article will be made available by the authors, without undue reservation.

## Author contributions

SGW: Funding acquisition, Supervision, Writing – original draft, Writing – review & editing. JL: Data curation, Formal Analysis, Resources, Visualization, Writing – original draft, Writing – review & editing. YW: Data curation, Formal Analysis, Resources, Visualization, Writing – review & editing. LZ: Data curation, Formal Analysis, Resources, Writing – review & editing. AZ: Formal Analysis, Visualization, Writing – review & editing. SSW: Resources, Writing – review & editing. YL: Resources, Writing – review & editing. DY: Resources, Writing – review & editing.
